# Point-of-Care Evaluation of Malaria Rapid Diagnostic Test (mRDT) for Detection of *Plasmodium falciparum* Among Children Under 5 Years of Age Attending Panyadoli Health Center III in Kiryandongo Refugee Settlement, Mid-Western Uganda

**DOI:** 10.1155/japr/9956261

**Published:** 2025-05-05

**Authors:** Dorcus Acan, Robert Opiro, Jacob Okot, Simon Peter Alarakol

**Affiliations:** ^1^Department of Biology, Faculty of Science, Gulu University, Gulu, Uganda; ^2^Laboratory Department, Kiryandongo General Hospital, Kiryandongo, Uganda; ^3^Gulu University Multifunctional Research Laboratories, Gulu, Uganda; ^4^Department of Biochemistry, Faculty of Medicine, Gulu University, Gulu, Uganda

**Keywords:** diagnostics, malaria, mid-Western Uganda, Panaydoli, refugees

## Abstract

**Background:** Malaria places a significant burden on Africa, accounting for 95% of global cases and 96% of malaria-related deaths, with children under five comprising 80% of these fatalities. Refugees and displaced persons face higher risks due to overcrowding and limited healthcare. The aim of this study was to compare the diagnostic performance of Malaria Rapid Diagnostic Test (mRDT) with microscopy among children under 5 years old who sought medical care at Panyadoli Health Center III in Panyadoli Refugee Settlement, Kiryandongo District, Uganda.

**Methods:** A cross-sectional study was conducted among refugee children under 5 years old from February to April 2023. A total of 380 blood specimens were obtained using the finger prick method and examined for malaria parasites using mRDT and microscopy. A structured questionnaire was used to collect sociodemographic characteristics of the respondents. Data were analyzed using descriptive statistics, while Kappa value was used to provide insights into the agreement between the two diagnostic methods.

**Results:** The prevalence of malaria among the study participants using mRDT and microscopy was 12.8% (95% CI: 8.0%–17.8%) and 12.2% (95% CI: 7.4%–17.4%), respectively. The sensitivity (Sn) and specificity (Sp) of mRDT were 94.5% (95% CI: 89%–98%), Sp at 94.0% (95% CI: 87%–98%), positive predictive value (PPV) at 92.0% (95% CI: 85%–96%), and negative predictive value (NPV) at 97.0% (95% CI: 93%–100%). The Sn and Sp of microscopy were 92.5% (95% CI: 87%–98%), Sp was 96.8% (95% CI: 91%–99%), PPV was 94.5% (95% CI: 89%–98%), and NPV was 97.5% (95% CI: 93%–99%). The overall kappa statistic (*κ*) for agreement between mRDT and microscopy was 0.75 (95% CI: 0.70–0.80), reflecting moderate to strong agreement between the two diagnostic methods.

**Conclusion:** The study found comparable malaria prevalence using mRDT and microscopy. Both methods showed high Sn and Sp with moderate to strong agreement, supporting mRDT's reliability in diagnosis. Due to its high accuracy and strong agreement with microscopy, mRDT can reliably diagnose malaria in resource-limited settings; however, confirmatory testing and periodic quality controls are recommended for accuracy and case management.

## 1. Introduction

Globally, 249 million malaria cases were estimated in 85 endemic countries in 2022 representing an increase of five million malaria cases as compared to 2021 [1]. In Uganda, there has been a general progress in reduction of malaria transmission from 42% in 2009 to 9% by 2018 [2]. However, the country was ranked the third-highest with global burden of malaria cases (5.1%), the seventh-highest level of deaths (3.2%) and the highest proportion of malaria cases in East and Southern Africa (23.2%) [3, 4]. Specifically, malaria affects over 90% of the population and is responsible for 20% of all hospital deaths [5]. According to the Ministry of Health (MOH), significant numbers of unreported deaths occur due to malaria at home, and 27.2% of all inpatient deaths among children < 5 years are attributed to malaria [5].

Diagnosis of malaria is one of the most important stages in the control of malaria infections in the endemic region [4]. However, this stage requires the use of fast, accurate, reliable, and cost-effective tools for use in resource-constrained settings before initiation of antimalarial treatment [4, 5]. According to the World Health Organization, all persons suspected of malaria infections should be confirmed using mRDT or microscopy before initiation of antimalarial treatment, underscoring the importance of these diagnostic techniques [2, 3]. Malaria Rapid Diagnostic Tests (mRDTs), microscopy, and polymerase chain reactions (PCRs) are robust diagnostic tools used in the diagnosis of malaria [6, 7]. mRDTs detect the presence of histidine rich proteins released from parasitized red blood cells and have been widely used for routine malaria diagnosis in many rural areas of sub-Saharan Africa [6]. However, its limited sensitivity among patients with low parasitaemia potentially makes it unreliable in the provision of effective clinical care to malaria patients [6, 7]. Traditionally, microscopy has been the most common diagnostic tool for the examination of blood smears for the presence of malaria parasites; however, it has low sensitivity among patients with low parasite densities [7–9], potentially contributing to delays in initiating necessary treatment, thus exacerbating the patients' conditions [6, 7]. Additionally, the extensive technical experiences required and significant delays associated with the staining procedures make it less reliable in the provision of timely clinical care for effective case management and treatment of malaria illness among patients [6, 9]. Despite the limitations, microscopy remains the primary gold standard for diagnosis of malaria among suspected patients [7, 9]. PCR, on the other hand, is a highly sensitive technique with a potential to detect patients with low parasite densities, but the costs, lengthy procedures, and extensive training required limit its use in many rural communities in developing countries [8, 9].

Uganda is the largest refugee hosting country in Africa, with over 1.36 million refugees [10–12]. Panyadoli Refugee Settlement Camp in Kiryandongo district is one of the largest refugee camps in the region, hosting refugees from South Sudan, Democratic Republic of Congo (DRC), Burundi, and Rwanda [10]. Panyadoli Health Center III provides healthcare services to most of the refugees in the settlement. The health facility has a Malaria Clinic that serves approximately 52,613 refugee population. This clinic uses mRDT and microcopy for routine Malaria diagnosis in the settlement and neighboring communities. Despite the extensive use of the two malaria diagnostics tools, their effectiveness in terms of diagnostic performance in diagnosis of malaria infections in the settlement has not been evaluated. The aim of this study was to evaluate the sensitivity and specificity of mRDT and compare it with that of microscopy among refugee children < 5 years old seeking medical care in the resource-constrained setting of Panyadoli Health Center III in Kiryandongo Refugees Settlement Camp in mid-Western Uganda.

## 2. Methods

### 2.1. Study Area

**Figure 1:**
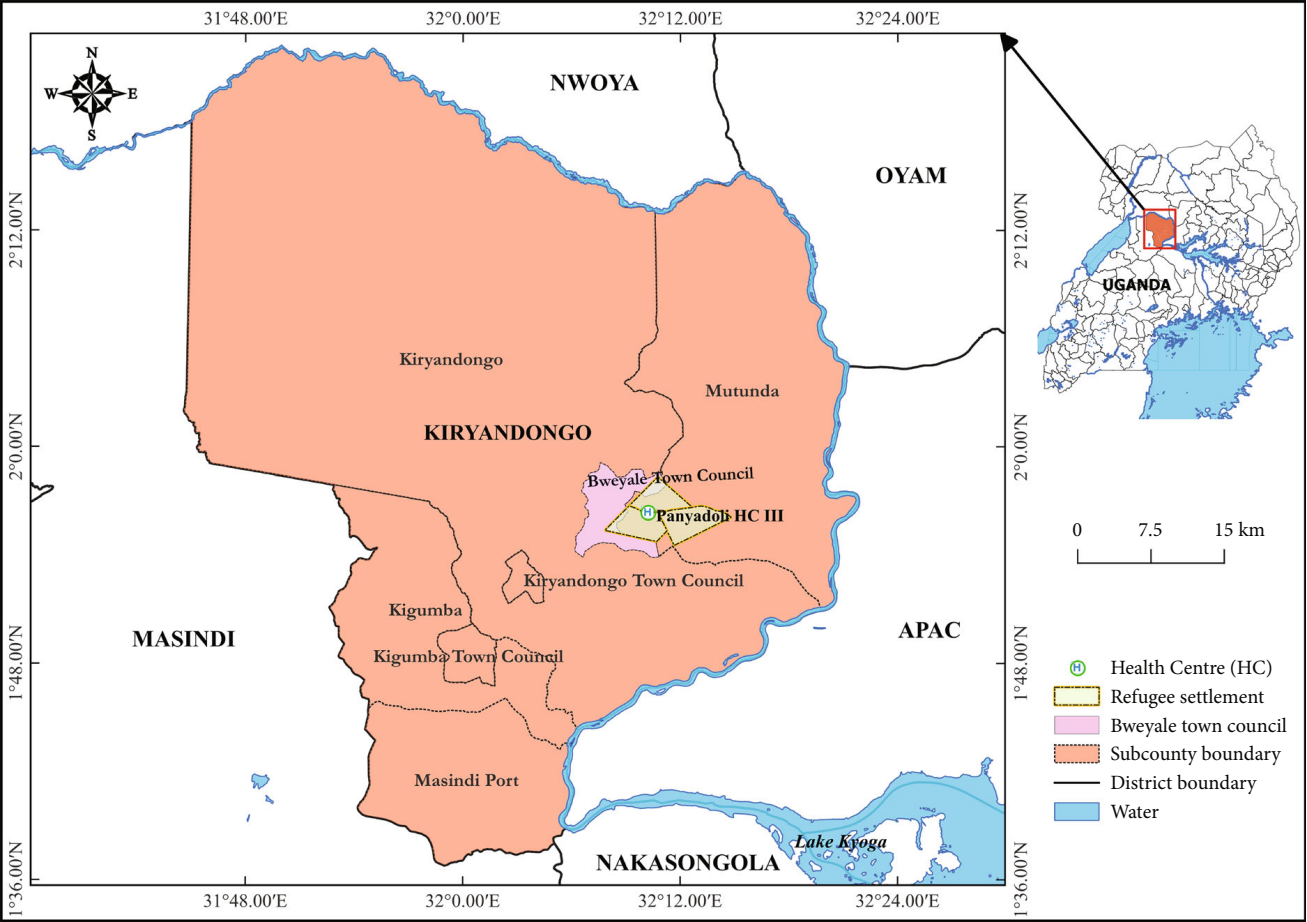
Map showing the location of Panyadoli Health Center III at Kiryandongo Refugee Settlement Camp in Midwestern Uganda.

The study was conducted in Panyadoli Health Center III situated in Kiryandongo Refugees Settlement Camp in Bweyale Town Council, Kiryandongo District (Figure 1). Kiryandongo District is located 255.5 km north of Kampala, the Capital City of Uganda, with coordinates 02 00 N, 32 18 E (latitude: 2.0000; longitude: 32.3000). The district's bimodal rainfall pattern creates a humid, wet climate that supports mosquito breeding and the growth of savannah woodland vegetation, which serves as both a vector habitat and a favorable environment for malaria transmission.

Kiryandongo district has a population of 280,000 people with an estimated 52,613 refugees [10]. Most of the refugees originated from South Sudan, with a few others from DRC, Kenya, Burundi, and Rwanda. A recent study at Panayadoli Refugee Settlement found a malaria prevalence of 12.6% among refugee children under 5 years old, with over 85% due to *Plasmodium falciparum* parasites [13]. At Kiryandongo General Hospital, 37.5% of children under five admitted with malaria had complicated cases, with the highest prevalence observed in the 2–3 years age group (25%) [14]. Panyadoli Health Center III is the only reliable source of health care services to the refugees and the host community. The health facility, like any other government entity in the region, grapples with limited funding for effective service delivery. The district, like others in the region, faces a shortage of skilled healthcare workers, making it challenging to provide adequate services to over 100,000 refugees and the host community. Village Health Teams (VHTs), government-trained community health workers, play a crucial role in primary healthcare, conducting household visits for disease surveillance, health education, and hygiene promotion.

### 2.2. Study Design and Study Population

This was a descriptive cross-sectional study where the malaria diagnostic performance of mRDT and microscopy was evaluated among young children at Kiryandongo Refugee Settlement Camp. Data on sociodemographic characteristics were also collected to enable examination of the causal relationships between these data and the performance outcomes of the two diagnostic tests. The study involved 380 refugee children under five with malaria symptoms, brought by parents/guardians for medical care at the health facility.

### 2.3. Sample Size Determination

Sample size of the participants was determined using Cochran's formula as described by [10]; *n*_o_ = *Z*^2^pq/*e*^2^, where *n*_o_ is the sample size, *Z* is the score at 95% confidence level giving a *Z* value of 1.96, derived from *Z*-table, *p* is the estimated proportion of the population with malaria among refugee children as previously reported by [10], and *e* is the desired level of precision (marginal error) = 0.05, *q* = 1 − *p*. Therefore, substituting this to the following formula: *n*_o_ = (1.96)^2^ × 0.5504 × 0.449/(0.05)^2^ = 380. So, *n*_o_ = 380.

### 2.4. Sampling Procedures

Suspected malaria patients presenting at Panyadoli Health Center III in Kiryandongo Refugee Settlement Camp were triaged and recorded in the register using the patients' identification number. Simple random sampling was then conducted with numbers assigned from a sampling frame of 700 based on the monthly malaria patients' records obtained from the outpatients malaria register. These numbers were aligned against the patients' identification number in the register, and any patient assigned a random number was included in the study. A total of 380 patients were selected and physically examined by a physician before referral to the laboratory for malarial tests. Parents or guardians who consented to their child's participation were given written informed consent forms, signed or thumb-printed, and interviewed. Those who declined still received necessary medical care. All malaria-positive children received treatment in line with the Uganda MOH guidelines, where uncomplicated malaria cases were treated with artemether/lumefantrine (AL), while severe cases received intravenous artesunate.

### 2.5. Inclusion and Exclusion Criteria

All eligible refugee children aged ≤ 5 years with suspected malaria symptoms, such as anorexia, vomiting, or abdominal discomfort, with or without diarrhea, were included. Additionally, patients with a body temperature above 37.4°C or a history of fever within the past 24–48 h were eligible. However, those seeking malaria confirmation or retesting after treatment were excluded from the study.

### 2.6. Specimen Collection and Examination

#### 2.6.1. mRDT

mRDT (Carestart, Lot no. 05CDH019A/05CDH030A) with a predetermined sensitivity and specificity of 97.89%–100% and 99.50%–100%, respectively, was used to detect the presence of malaria parasites in the patients' blood. Briefly, the test device and the sample pipette were removed from the foil pouch. The test device was labeled with the patient's identification number and placed on a flat surface. Using the provided micropipette, 5 *μ*L of whole blood sample was obtained from a finger prick and added to the sample well of the test card. Two drops (60 *μ*L) of assay diluent were added into the well and allowed to flow by capillary action. The test result was read within 15–20 min and recorded as positive or negative depending on the test outcomes.

#### 2.6.2. Microscopic Examination of Malaria Parasites

The diagnosis of malaria was done on patients' samples using a microscope according to the standard operating procedures recommended by [1]. Briefly, a clean glass slide was labeled with the patient's assigned unique number and accessioned in the register. The third finger from the thumb was cleaned with 70% alcohol swab and left to air dry. The blood specimen was collected using a sterile lancet upon consent from the parents/guardians of the children. Thick blood smears were then prepared by placing a drop of whole blood on a clean glass slide labeled with the patients' assigned number. A blood film measuring at least two diameters was then spread onto the glass slide and air-dried. This was followed by dipping in Field Stain A for 5 s and washed to remove excess stains. The slide was then dipped in Field Stain B for 10 s and washed in clean water followed by air-drying. The thin smear was prepared by placing a drop of whole blood at the edge of a labeled glass slide and then spread forward using a cover slip to around 10 diameters. The film was air-dried, fixed using absolute methanol for 1 min, stained with Field Stain B for 10 s, and washed in clean water. The slide was then dipped in Field Stain A for 15–30 s and washed in clean water. The back of the slide was wiped with clean cotton wool and allowed to dry vertically. The stained slides were examined on a microscope using X100 magnification lenses for both smears and X40 for thin smears. Examination for the film was done for at least 10 min (approximately 200 oil immersion fields), before declaring the slide negative.

### 2.7. Data Analyses

Data was analyzed in IBM SPSS Statistics (Version 26.0) using descriptive statistics and displayed via frequencies and percentages. The prevalence rate of *Plasmodium falciparum* was calculated using the formula: *P* = *d*/*N*∗100, where *d* is the number of under five children positive for *Plasmodium falciparum* parasites and *N* is total number of under five children examined for *Plasmodium falciparum*. Diagnostic performance of both mRDT and microscopy was evaluated using latent class analysis (LCA) [15], as we do not assume either test is a perfect gold standard. LCA was performed to estimate the true malaria prevalence, sensitivity, specificity, positive predictive value (PPV), negative predictive value (NPV), and kappa coefficient for both mRDT and microscopy. LCA allows for the estimation of a latent disease status, which was used as the “true” malaria status to assess the performance of the two diagnostic tests. The model was fitted using the poLCA package [16] in R [17], which calculates the posterior probabilities of the latent classes (malaria positive vs. malaria negative) based on the test results. Both tests were analyzed simultaneously to account for their agreement and discordance. We assessed the agreement between mRDT and microscopy by calculating the kappa coefficient, which quantifies the level of agreement beyond chance. The model assumed no gold standard and used the test outcomes of mRDT and microscopy as indicators for the latent classes of disease. The estimated diagnostic performance metrics were based on these class probabilities, and the kappa statistic was used to measure the level of agreement between mRDT and Microscopy. *p* values less than 0.05 were considered statistically significant at a 95% confidence interval.

## 3. Results

### 3.1. Sociodemographic Information of Participants

Overall, a total of 380 refugee children aged between 3 and 59 months were enrolled in this study. A majority of 111 (29.2% [95% Cl: 24.2–43.2] were in the age group of 3–12 months (Table 1). Out of the 380 refugee children enrolled, there were 187 (49.2% [95% Cl: 44.2–54.2]) males and 193 (50.8% [95% Cl: 45.8–54.8]) female, respectively. A majority of 232 (61.1% [95% Cl: 55.1–66.1]) of the refugee children were living in households having between 1 and 9 members, while 148 (38.9% [95%Cl: 33.9–43.2]) households had between 10 and 36 members.

### 3.2. Diagnostic Performance of mRDT and Microscopy

The estimated prevalence of malaria, as derived from the latent classes, was 12.8% (95% CI: 8.0%–17.8%) and 12.2% (95% CI: 7.4%–17.4%) as per as per the mRDT and microscopy, respectively. For mRDT, the sensitivity was estimated at 94.5% (95% CI: 89%–98%), specificity at 94.0% (95% CI: 87%–98%), PPV at 92.0% (95% CI: 85%–96%), and NPV at 97.0% (95% CI: 93%–100%). For microscopy, the sensitivity was 92.5% (95% CI: 87%–98%), specificity was 96.8% (95% CI: 91%–99%), PPV was 94.5% (95% CI: 89%–98%), and NPV was 97.5% (95% CI: 93%–99%). The overall kappa statistic for agreement between mRDT and microscopy was 0.75 (95% CI: 0.70–0.80), reflecting moderate to strong agreement between the two diagnostic methods (Table 2).

### 3.3. RDT and Microscopy Positivity in Relation to Sociodemographic Characteristics

Table 3 presents the results of malaria tests among under five refugee children using microscopy and mRDT. Malaria positivity detected by mRDT increased with age, with a mean age of 36 months. The highest number of malaria cases was observed in children aged 48–60 months (17 cases, 4.5% [95% CI: −0.5–9.5]), followed by those aged 26–47 months (12 cases, 3.24% [95% CI: −1.76–8.24]), and children under 12 months (9 cases, 2.4% [95% CI: −2.6–7.4]). The lowest malaria prevalence was in children aged 12–23 months (4 cases, 1.1% [95% CI: −3.9–6.1]). Malaria positivity detected by mRDT was higher in female children (33 cases, 8.7% [95% CI: 3.7–13.7]) than males (17 cases, 4.5% [95% CI: −0.5–9.5]). Additionally, malaria prevalence increased with household size. Children from households with 10–36 members had more cases (30 cases, 7.9% [95% CI: 2.9–12.9]) compared to those from households with 1–9 members (18 cases, 4.7% [95% CI: −0.3–9.7].

## 4. Discussion

This study is aimed at evaluating the sensitivity and specificity of mRDT compared to microscopy in diagnosing malaria among children under 5 years old at Panyadoli Health Center III, Kiryandongo Refugee Settlement, in mid-Western Uganda. The malaria prevalence was 12.8% using mRDT and 12.2% with microscopy, indicating that mRDT yields comparable results to microscopy. Given its high accuracy, mRDT can play a crucial role in diagnosing malaria among vulnerable refugee children in the region. As the refugee population in Panyadoli grows, mRDT is well positioned to be a key tool in providing timely and routine malaria care, as both mRDT and microscopy are primary diagnostic methods in local health facilities.

The results from mRDT and microscopy indicate a moderate average malaria prevalence of around 12.5%, which is relatively low compared to other studies conducted in the East African region, where malaria prevalence remains higher. For instance, a study among children under five in Kinshasa, DRC, reported a prevalence of 17.0% [12]. Similarly, a study in Kirehe District, Eastern Province of Rwanda, found a malaria prevalence of 19.7% among children under five [13]. Other studies also reported higher prevalence rates [14–16]. In contrast, a study in Federal Medical Centre, Asaba, Nigeria, found a higher prevalence of 53% among children under five [7], while another in Kano, Northern Nigeria, reported 67% [17]. The low malaria prevalence observed in this study can be attributed to effective malaria control interventions; refugee settlements in Uganda benefit from targeted programs like insecticide-treated nets, indoor residual spraying, and active case detection, which reduce transmission.

The results showed similar performance for mRDT and microscopy, with mRDT having sensitivity of 94.5% and specificity of 94%, while microscopy had 92.5% sensitivity and 96.8% specificity. The high accuracy may be due to the use of a high-quality histidine-rich protein 2 (HRP2) RDT (sensitivity of 97.89%–100%; specificity of 99.50%–100%) and trained technicians. These findings demonstrate mRDT's excellent performance, consistent with studies from Northwest Ethiopia, which reported 93.2% sensitivity and 98.6% specificity [12, 18]. However, it contrasts with studies in low-transmission areas, where mRDT sensitivity and specificity were lower at 83.3% and 96%, respectively [13, 19]. Similarly, other studies reported reduced sensitivity and specificity, such as 87.74% and 89.1% [6] and 83.7% and 89.1% [5]. A study in Ghana found an even lower sensitivity (39.3%) and specificity (55.7%) but high PPV (95.7%) and NPV (94.5%) [7]. Variations in diagnostic accuracy may stem from differences in staff expertise [5, 7], mRDT types, methodologies, and factors like false positives and negatives affecting result estimation.

The high sensitivity of mRDT suggests that it is effective for routine malaria diagnosis in refugee settlements. Moreover both mRDT and microscopy showed similar specificities (94% vs. 96%), indicating their reliability in diagnosing suspected malaria cases in children under five. Moreover, the strong agreement between mRDT and microscopy (Kappa = 0.75) further confirms the effectiveness of mRDT as a diagnostic tool. The positive and NPVs provided additional insights into the findings. mRDT had a 92% PPV, slightly less than that of microscopy (94.5%), and both had a NPV of around 97%. These results emphasize the accuracy of both methods in detecting *Plasmodium falciparum*, supporting their use in resource-limited settings like refugee settlements. However, their limitations in detecting low parasite densities are well documented [5].

Sensitivity and specificity in mRDT detection increased with age, which is consistent with the understanding that older children tend to have higher parasite densities due to repeated malaria exposure, which enhances the detection of *Plasmodium falciparum* by RDTs [8]. Older children are more likely to have higher parasitemia, making it easier for tests to identify the presence of malaria antigens, such as HRP2, which is the target of many malaria RDTs [18]. Studies have shown that mRDT sensitivity and specificity tend to improve as parasite load increases, which is more common in older children [8].

## 5. Conclusion

The diagnostic performance of mRDT was comparable to microscopy, making it a reliable tool for the rapid diagnosis of malaria in resource-limited health facilities, such as refugee settlements. There was a strong positive agreement between mRDT and microscopy in detecting malaria parasites among refugee children under five at Panyadoli Refugee Settlement. mRDT can be effectively used for rapid malaria diagnosis in routine clinical care, while microscopy remains valuable for confirmatory testing when needed. However, its use should be guided by clinical assessments from healthcare providers to ensure accurate diagnosis and management.

### 5.1. Limitations of the Study

This study used only mRDT and microscopy for detecting malaria in children under five, and a more sensitive method like PCR could have improved comparisons. The small sample size may have also limited accuracy, and the focus on children restricts applicability to other age groups. Additionally, the absence of malaria parasite density measurements limited assessment of mRDT performance across parasitemia levels. Future studies should include quantitative microscopy or molecular methods to stratify diagnostic performance by parasite burden. Despite these limitations, the findings support mRDT as a reliable diagnostic tool in resource-limited refugee settlements in mid-Western Uganda.

## Figures and Tables

**Table 1:** Sociodemographic characteristics of participants.

**Variables**	**Category**	**Frequency**	**Percentage**	**Mean age**	**Std**
Gender	Female	187	49.2	—	—
	Male	193	50.8	—	—

Age group	3–12	111	29.2	—	—
	13–24	65	17.1	—	—
	25–36	50	13.2	36	15.4
	37–48	53	13.9	—	—
	49–59	62	16.3	—	—

House hold sizes	1–9	232	69.9	—	—
	10–36	118	31.1	—	—

*Note:* Std, standard deviation.

**Table 2:** Performance of accuracy of mRDT and microscopy.

**Diagnostic method**	**Sensitivity (%)** **95% CI**	**Specificity (%)** **95% CI**	**PPV (%)** **95% CI**	**NPV (%)** **95% CI**	**Kappa statistic** **95% CI**
mRDT	94.5 (89–98)	94 (87–98)	92 (85–96)	97 (93–100)	0.75 (0.70–0.80)
Microscopy	92.5 (87–98)	96.8 (91–99)	94.5 (89–98)	97.5 (93–99)	

**Table 3:** Relationships between the sociodemographic characteristics and the diagnostic tests.

**Variable category**		**Microscopy**				**X** ^2^	**p**	**RDT**				**X** ^2^	**p**
		**Negative**		**Positive**				**Negative**		**Positive**			
Sex	Male	171	(45.0%)	16	(4.2%)	5.541	0.019⁣^∗^	170	(44.7%)	17	(4.5%)	5.330	0.021⁣^∗^
	Female	161	(42.4%)	32	(8.4%)			160	(42.1%)	33	(8.7%)		

Age group	> 12	102	(26.8%)	9	(2.4%)	13.84	0.008⁣^∗^	102	(26.8%)	9	(2.4%)	13.673	0.008⁣^∗^
	12–23	65	(17.1%)	3	(0.8%)			64	(16.8%)	4	(1.1%)		
	24–35	50	(13.2%)	7	(1.8%)			50	(13.2%)	7	(1.8%)		
	36–47	53	(13.9%)	12	(3.2%)			53	(13.9%)	12	(3.2%)		
	48–60	62	(16.3%)	17	(4.5%)			61	(16.1%)	18	(4.7%)		

Religion	Catholic	226	(59.5%)	33	(8.7%)	2.693	0.610	224	(58.9%)	35	(9.2%)	2.683	0.612
	Anglican	75	(19.7%)	13	(3.4%)			75	(19.7%)	13	(3.4%)		
	Muslim	12	(3.2%)	0	(0.0%)			12	(3.2%)	0	(0.0%)		
	Born again	15	(3.9%)	2	(0.5%)			15	(3.9%)	2	(0.5%)		
	SDA	4	(1.1%)	0	(0.0%)			4	(1.1%)	0	(0.0%)		

Education level (parent/guardian)	Nonformal	306	(80.5%)	40	(10.5%)	5.084	0.079	304	(80.0%)	42	(11.1%)	4.545	0.103
	Primary	20	(5.3%)	5	(1.3%)			20	(5.3%)	5	(1.3%)		
	Secondary	6	(1.6%)	3	(0.8%)			6	(1.6%)	3	(0.8%)		
	Tertiary	0	(0.0%)	0	(0.0%)			0	(0.0%)	0	(0.0%)		

Country of origin	South Sudan	320	(84.2%)	46	(12.1%)	0.770	0.857	318	(83.7%)	48	(12.6%)	0.725	0.867
	Rwanda	5	(1.3%)	1	(0.3%)			5	(1.3%)	1	(0.3%)		
	Kenya	3	(0.8%)	0	(0.0%)			3	(0.8%)	0	(0.0%)		
	Congo	4	(1.1%)	1	(0.3%)			4	(1.1%)	1	(0.3%)		

Household size group	1–9	214	(56.3%)	18	(4.7%)	0.070	0.791	213	(56.1%)	31	(8.2%)	0.122	0.726
	10–36	118	(31.1%)	30	(7.9%)			117	(30.8%)	19	(5.0%)		

⁣^∗^Significant values (*p* < 0.05).

## Data Availability

The data that support the findings of this study is available on request from the corresponding author. The data are not publicly available due to privacy or ethical restrictions.

## Nomenclature

mRDT: Malaria Rapid Diagnostic Test
PPV: positive predictive value
NPV: negative predictive value
PCR: polymerase chain reaction
VHTs: village health teams
TP: true positive
TN: true negative
FP: false positive
FN: false negative
CI: confidence interval
